# Investigation of nitrogen and phosphorus contents in water in the tributaries of Danjiangkou Reservoir

**DOI:** 10.1098/rsos.170624

**Published:** 2018-01-24

**Authors:** Yan Liu, Yuanyuan Zhu, Xiaocui Qiao, Binghui Zheng, Sheng Chang, Qing Fu

**Affiliations:** 1 State Key Laboratory of Environmental Criteria and Risk Assessment, State Environmental Protection Key Laboratory of Drinking Water Source Protection, Chinese Research Academy of Environmental Sciences, Beijing 100012, People's Republic of China; 2 Chinese Academy of Environmental Planning, Beijing 100012, People's Republic of China

**Keywords:** Danjiangkou Reservoir, tributaries, nitrogen, phosphorus, nutrient structure, correlation

## Abstract

As part of the efforts to ensure adequate supply of quality water from Danjiangkou Reservoir to Beijing, surface water samples were taken from the tributaries of Danjiangkou Reservoir in the normal (May), flood (August) and dry (December) seasons of 2014, and characterized for nitrogen and phosphorus contents as specified in the applicable standards. Test results indicated that (i) the organic pollution in the Sihe and Shendinghe rivers was more serious than those in other tributaries, and the concentrations of nitrogen and phosphorus favoured the growth of most algae; (ii) total phosphorus (TP), total nitrogen (TN) and dissolved inorganic nitrogen (DIN) were in the forms of dissolved phosphorus (DTP), dissolved nitrogen (DTN) and nitrate nitrogen $\left({\mathrm{NO}}_{3}^{-}\text{-N}\right)$, respectively, in these seasons; (iii) compared with nitrogen, phosphorus was more likely to block an overrun of phytoplankton; (iv) TN, TP, permanganate index (COD_Mn_) and other ions were positively correlated. These findings are helpful for the government to develop effective measures to protect the source water in Danjingkou Reservoir from pollution.

## 1. Introduction

Danjiangkou Reservoir has become increasingly popular because it is the headwaters for China's programme of diverting water from central China to guarantee adequate supply of quality water to more than twenty cities in North China including Beijing, Tianjin and Shijiazhuang [1,2]. A population of 110 million people over an area of 15.1 × 10^4^ square kilometres have benefited from the programme. The supply of water from Danjiangkou Reservoir to Beijing officially started on 12 December 2014. To ensure adequate supply of quality water to Beijing, it is of great significance to maintain a good ecological environment at the reservoir and to ensure good water and soil conservation in its upper reaches. The nitrogen and phosphorus in water in the reservoir come from its tributaries [3,4], and hence, the most effective measures to prevent and cure algae bloom and water eutrophication is to control the total amount of pollutants in each of its tributaries. Much work has been done on this particular aspect in recent years. For example, water pollution risks in the region of Shiyan were assessed using the proposed model [5]. Necessity and operational feasibility of early reservoir refill were analysed [6]. An eco-environmental vulnerability assessment method was developed using an environmental information system database for the Danjiangkou Reservoir Area [7]. The spatio-temporal changes of the carbonate system and CO_2_ flux of a hydroelectric reservoir (Danjiangkou Reservoir) were studied in the subtropical monsoon climate region [8]. The spatial distributions of Cd, Pb, Cu, Zn, Cr and As in soil of the relocation areas of Danjiangkou Reservoir were analysed and the degrees of contamination and potential ecological risk of these elements were evaluated using the integrated pollution index and the potential ecological risk index [9]. The possibility for preventing downstream diatom blooms was explored using the water storage in the Danjiangkou Reservoir and a flushing strategy was developed to control diatom growth [10]. A functional group approach was used on the basis of the Q index to analyse the spatial and temporal patterns, environmental factors and the ecological status of phytoplankton in the Danjiangkou Reservoir of China from July 2011 to April 2012 [11]. However, little work has been done on the nitrogen and phosphorus contents in water in the tributaries of Danjiangkou Reservoir.

Therefore, as part of the efforts to guarantee adequate supply of quality water to Beijing, surface water was sampled from the major tributaries of Danjiangkou Reservoir in the normal (May), flood (August) and dry (December) seasons of 2014, and characterized for nitrogen and phosphorus contents. These findings of the investigation provided an important basis for the effective measures taken later on as appropriate to ensure adequate supply of quality water to Beijing.

## 2. Material and methods

### 2.1. Sampling and characterization

Danjiangkou Reservoir is located at 32°36′–33°48′ N, 110°59′–111°49′ E [12]. It has a surface area of about 1050 square kilometres, an average annual precipitation of 800–1000 mm and an annual mean air temperature of 14.4–15.7°C. The reservoir has a normal storage level of 170 m, a storage capacity of 290.5 billion cubic metres and a shoreline of 4610.6 km [13,14].

**Figure 1.**
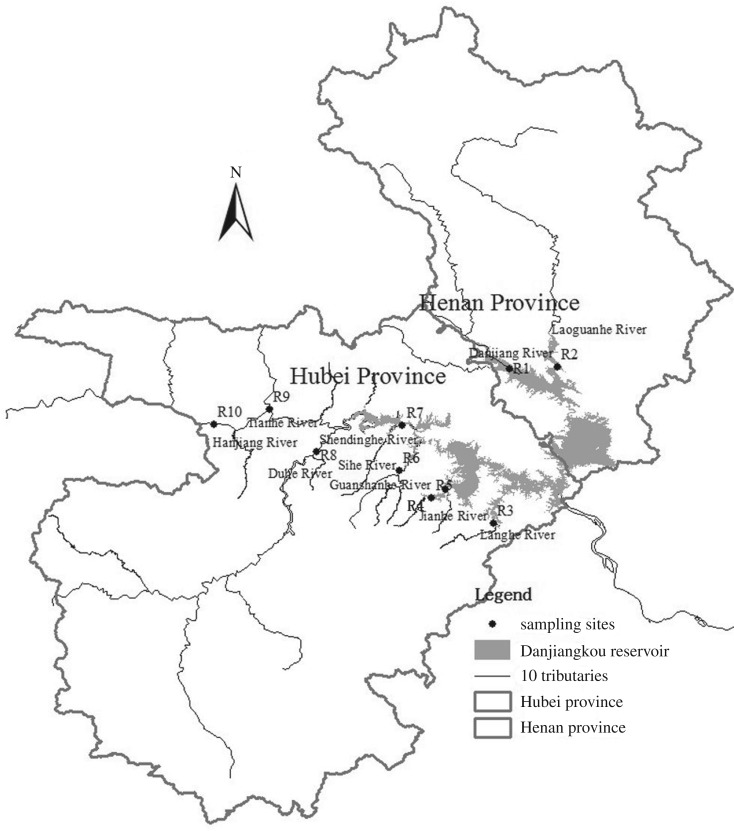
Locations of 10 sampling points.

It can be seen from figure 1 that the reservoir has 10 major tributaries including the Danjiang (33°1′ N, 111°18′ E), Laoguanhe (33°1′ N, 111°29′ E), Langhe (32°25′ N, 111°14′ E), Jianhe (32°33′ N, 111°3′ E), Guanshanhe (32°31′ N, 111°0′ E), Sihe (32°37′ N, 110°53′ E), Shendinghe (32°48′ N, 110°53′ E), Duhe (32°42′ N, 110°34′ E), Tianhe (32°51′ N, 110°23′ E) and Hanjiang (32°48′ N, 110°11′ E) rivers. Sampling points sequenced from number R1 to R10. Surface water was sampled in the normal (May), flood (August) and dry (December) seasons of 2014, and no permissions were required prior to conducting our fieldwork. Two litres of surface water were collected at a depth of 50 cm using a Van Dorn bottle. The samples were stored in polyethylene bottles and then transported to the laboratory in a cooler at 4°C with a maximum storage time of one week [15,16]. The measurements of water quality covered total nitrogen (TN), nitrate nitrogen $\left(\text{N}{{\text{O}}_{3}}^{-}\text{-N}\right)$, ammonia nitrogen $\left(\text{N}{\text{H}}_{3}\text{-N}\right)$, dissolved nitrogen (DTN), total phosphorus (TP), dissolved phosphorus (DTP), water temperature (T), pH, dissolved oxygen (DO) and electrical conductivity (EC), 5 days' biochemical oxygen demand (BOD_5_), permanganate index (COD_Mn_), fluoride (F^−^), sulfate $\left(\text{S}{{\text{O}}_{4}}^{2-}\right)$ and chloride (Cl^−^).

### 2.2. Methods

After collecting surface water, one litre of surface water was first filtered using a 0.45 µm microporous membrane, and then sulfuric acid (1 mol l^−1^) was added immediately to preserve it before the concentrations of DTN, $\text{N}{{\text{O}}_{3}}^{-}\text{-N}$, NH_3_-N and DTP were measured. Another half litre of surface water was first filtered using a 0.45 µm microporous membrane before the concentrations of F^−^, $\text{S}{{\text{O}}_{4}}^{2-}$ and Cl^−^ were measured. Sulfuric acid (1 mol l^−1^) was added to the last half litre of surface water before the concentrations of TN, TP, COD_Mn_ and BOD_5_ were measured. T, pH, DO and EC were measured using a multi-parametric probe YSI6600 on site. The chemical parameters were measured as required in the specification for ‘analysis in water and wastewater’. The titration method was used to determine the concentration of COD_Mn_. The concentration of BOD_5_ was determined using the dilution and inoculation method. Nessler's reagent colorimetric method was used to measure the concentration of NH_3_-N. The concentrations of DTP and TP were determined by the ammonium molybdate spectrophotometric method. The concentrations of DTN and TN were determined by the alkaline potassium persulfate digestion ultraviolet spectrophotometer method. Chromatography of ions was used to determine the concentration of $\text{N}{{\text{O}}_{3}}^{-}\text{-N}$ [17]. All analyses were carried out and documented in triplicate with mean values used for subsequent data processing. The concentration of particulate nitrogen (PN) was obtained by subtracting the concentration of DTN from the concentration of TN. The concentration of NH_3_-N plus the concentration of $\text{N}{{\text{O}}_{3}}^{-}\text{-N}$ equals the concentration of dissolved inorganic nitrogen (DIN). The concentration of particulate phosphorus (PP) was obtained by subtracting the concentration of DTP from the concentration of TP [18,19].

For the present study, the annual average water quality parameter was given by

2.1 $$C=\frac{C_{f}\times 3+C_{n}\times 4+C_{d}\times 5}{12},$$

where *C* is the annual average content (mg l^−1^), *C*_f_ is the content in the flood season (mg l^−1^), *C*_n_ is the content in the normal season (mg l^−1^) and *C*_d_ is the content in the dry season (mg l^−1^).

Statistical analyses were performed with SPSS (v. 17.0) [20], Excel 2007 and Origin (v. 8.0). The data were standardized to eliminate the influence of dimension during the cluster analysis.

The inputs of TN and TP were given by

2.2 $$W_{i}=31.536\times 10^{-2}\times Q_{i}\times C_{i},$$

where *W_i_* is the input of TN and TP of tributary *i* (t a^−1^), *Q_i_* is the annual average discharge of tributary *i* (m^3^ s^−1^), which was provided by Nanyang and Shiyan Hydrology Bureaus, and *C_i_* is the average annual TN and TP content of tributary *i* (mg l^−1^).

### 2.3. Water quality standard

According to Surface Water Environmental Quality Standard of China (GB3838-2002), the specific standards of water quality parameters are given in table 1.

**Table 1.** Environmental Quality Standard (GB3838-2002) for Surface Water in units of mg l^−1^ (pH without unit).

water quality parameters	I	II	III	IV	V
pH		6–9			
Cl^−^		≤ 250			
$\text{S}{{\text{O}}_{4}}^{2-}$		≤ 250			
F^−^	≤ 1.0			≤ 1.5	
BOD_5_	≤3	≤3	≤4	≤6	≤10
COD_Mn_	≤2	≤4	≤6	≤10	≤15
TP	≤0.02	≤0.1	≤0.2	≤0.3	≤0.4
TN	≤0.2	≤0.5	≤1	≤1.5	≤2

## 3. Results and discussion

### 3.1. Parameter contents in water in tributaries of Danjiangkou Reservoir

#### 3.1.1. Physical and chemical characteristics

**Figure 2.**
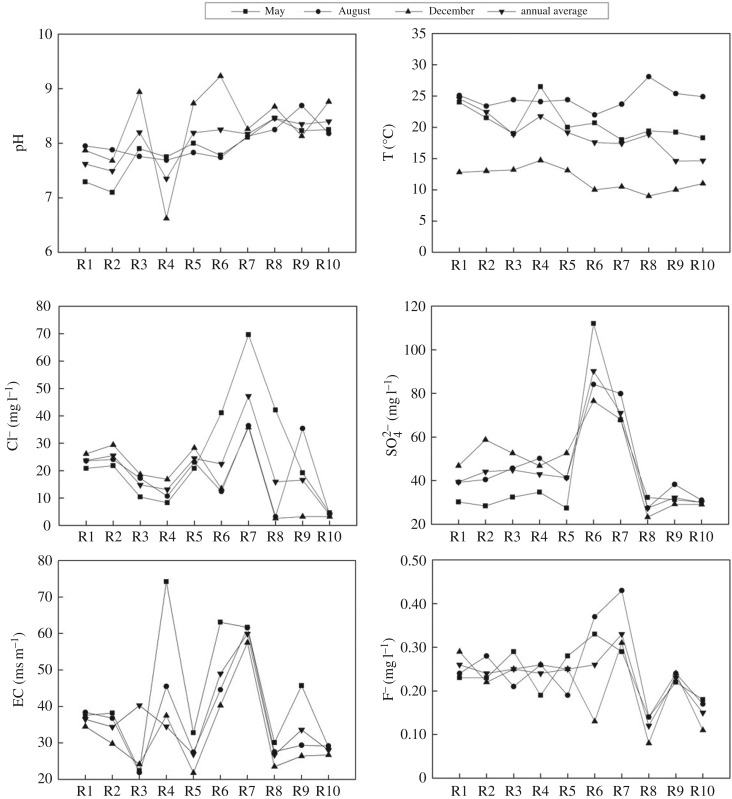
Physical and chemical characteristics of water in the tributaries.

As shown in figure 2, the annual average value of pH is in the range of 7.35–8.46, which implies a weak alkalinity. The water temperature is in the range of 9–14.7°C in the dry season. It can be seen through comparison that there is a quick increase in temperature in the normal and flood seasons. Water bloom is more likely to occur because the temperature in normal and flood seasons accelerates the growth of phytoplankton (18–25°C). The annual average content of Cl^−^ and $\text{S}{{\text{O}}_{4}}^{2-}$ are far below the surface water environmental quality standard (GB3838-2002) where the regulation limits are 250 mg l^−1^ for tributaries. The annual average contents of $\text{S}{{\text{O}}_{4}}^{2-}$ in Sihe River (R6) and Shendinghe River (R7) are higher than that in other tributaries. The annual average content of Cl^−^ in Shendinghe (R7) River is higher than that in other tributaries. The annual average value of EC is in the range of 26.7–59.9 mS m^−1^. The annual average content of F^−^ is in the range of 0.12–0.33 mg l^−1^, which is below the grade I standard of water quality standard GB3838-2002. The contents of other ions are good.

#### 3.1.2. Characteristics of organic pollutants

**Figure 3.**
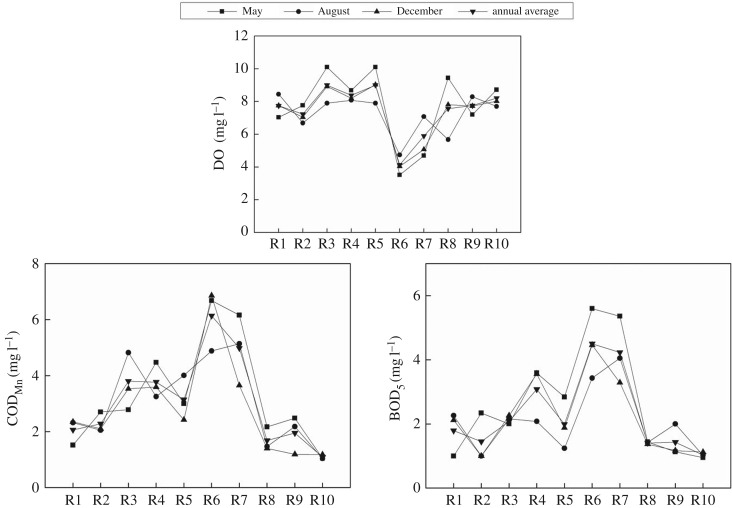
DO, BOD_5_ and COD_Mn_ in water in the tributaries.

As shown in figure 3, the annual average content of DO is in the range of 4.13–9.00 mg l^−1^. As a whole, the tributaries are rich in DO. The contents of DO in the flood season are lower than that of the normal and dry seasons, which is possibly due to the facts that (i) the high temperature in August reduces the surface pressure of water; (ii) the microbial activity and organic pollutants consume large amounts of DO. The annual average contents of BOD_5_ and COD_Mn_ are in the range of 1.03–4.50 mg l^−1^ and 1.09–6.14 mg l^−1^, respectively. BOD_5_ contents in Jianhe River (R4, in December and May), Sihe River (R6) and Shendinghe River (R7) are higher than the grade II standard (3 mg l^−1^) of water quality standard GB3838-2002, the annual average contents of which are about 3.08, 4.50 and 4.23 mg l^−1^, respectively. Therefore, the organic pollution in Sihe River is the most serious.

#### 3.1.3. Characteristics of nitrogen pollutant

**Figure 4.**
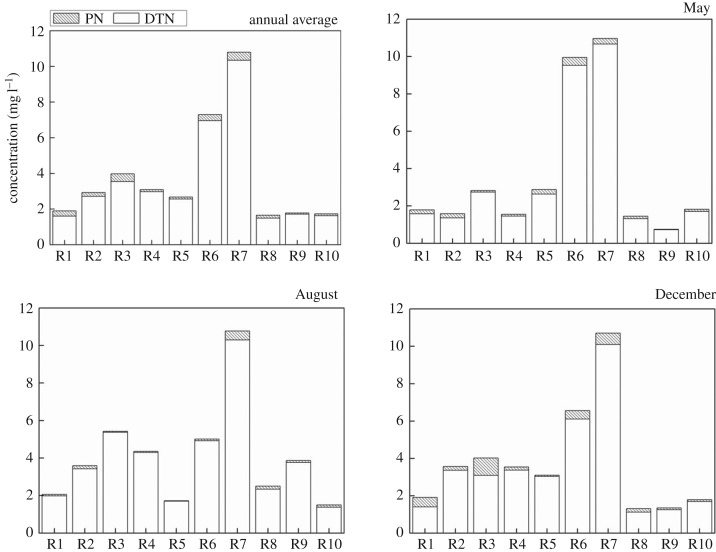
PN and DTN in water in the tributaries.

As shown in figure 4, the annual average content of TN is in the range of 1.65–10.81 mg l^−1^. The highest annual average content of TN at 10.81 mg l^−1^ is found in Shendinghe River, followed by Sihe River which has an annual average content of 7.30 mg l^−1^. Nitrogen pollution in Sihe River and Shendinghe River are more serious compared with that in other tributaries. It also could be found that TN in Shendinghe River changes little among seasons because the nitrogen in Shendinghe River mainly comes from urban sewage, industrial wastewater and other point sources in Shiyan City, and the emissions from these point sources are more stable and less affected by rainfall.

**Figure 5.**
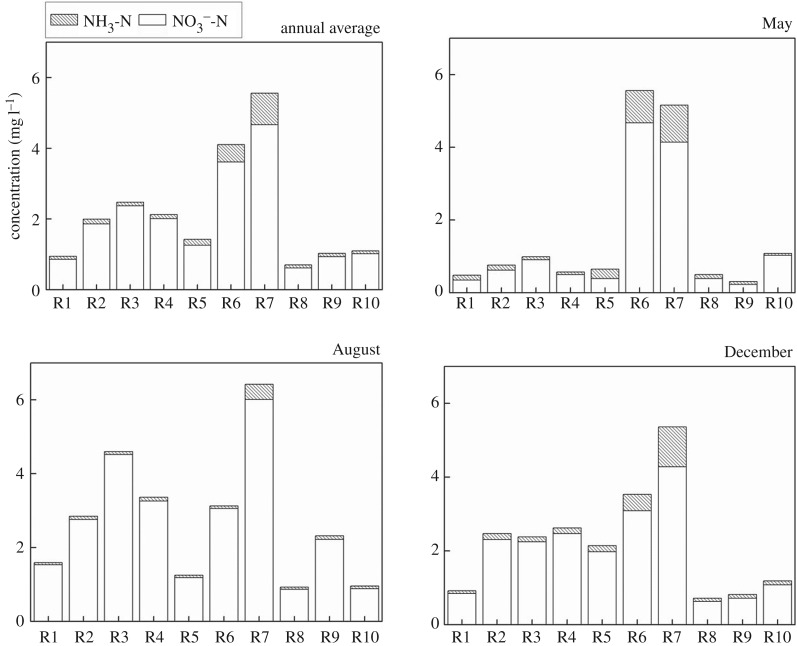
NH_3_-N and $\text{N}{{\text{O}}_{3}}^{-}\text{-N}$ in water in the tributaries.

As shown in figure 5, the percentage of DIN in TN is more than 50% in the tributaries of Danjiangkou Reservoir. DIN is the dominant form of nitrogen and high DIN ensures the full utilization of nitrogen. The annual average concentration of $\text{N}{{\text{O}}_{3}}^{-}\text{-N}$ is in the range of 0.61–4.67 mg l^−1^, which accounts for more than 90% in DIN in all tributaries, while the percentage of NH_3_-N in DIN is less than 10%. NH_3_-N is the reduced form of nitrogen and $\text{N}{{\text{O}}_{3}}^{-}\text{-N}$ is the steady form of nitrogen. Generally, nitrogen is discharged into the water in the reduced form. NH_3_-N is oxidized to $\text{N}{{\text{O}}_{2}}^{-}\text{-N}$, and then oxidized to $\text{N}{{\text{O}}_{3}}^{-}\text{-N}$ after nitrification. Such a nitrification process would consume a large amount of oxygen (4.57 mg mg^−1^) [21,22]. According to the percentage of $\text{N}{{\text{O}}_{3}}^{-}\text{-N}$ and NH_3_-N, old pollutants have been broken down, but there are still new pollutants being discharged into the water. In the flood season, the proportion of $\text{N}{{\text{O}}_{3}}^{-}\text{-N}$ in TN is higher than that in other seasons, while the proportion of NH_3_-N in TN decreased, because the higher temperature in the flood season is good for nitrification.

#### 3.1.4. Characteristics of phosphorus pollutant

**Figure 6.**
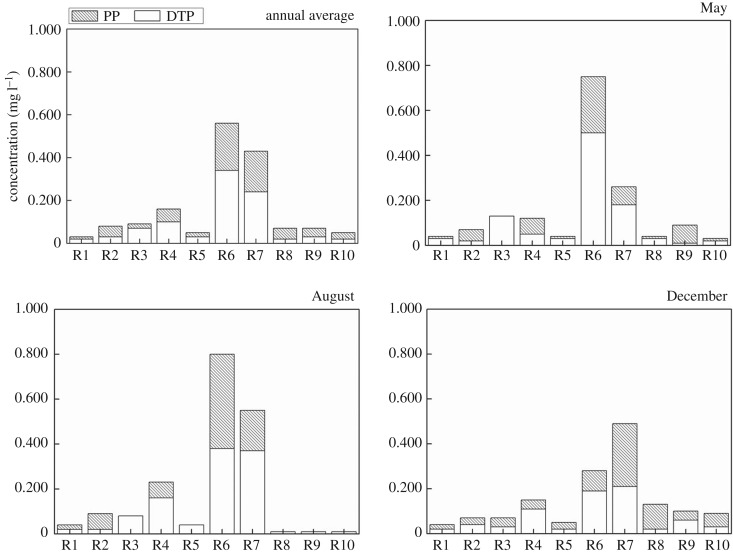
PP and DTP in water in the tributaries.

As shown in figure 6, the annual average content of TP is in the range of 0.03–0.56 mg l^−1^, followed by Shendinghe River which has an annual average content of 0.43 mg l^−1^. The annual average contents of TP in Sihe River and Shendinghe River are both higher than the grade III standard (0.2 mg l^−1^), which means that phosphorus pollution in Sihe and Shendinghe Rivers is more serious than that in other tributaries. This finding is in agreement with previous reported works [23,24]. The two rivers receive huge amounts of wastewater from urban sewage and other point sources in Shiyan City. The use of phosphorus detergent is also a major cause for the high TP content. In addition, the field survey showed that there is a small amount of phosphate fertilizers used in Shiyan city and these plants may also be an important cause for the high TP content.

The annual average content of PP is in the range of 0.01–0.22 mg l^−1^. The percentage of PP in TP is 22.22–71.43%. The percentage of PP in TP is more than 50% in Laoguanhe River, Duhe River, Tianhe River and Hanjiang River, which implies that PP is the main phosphorus form in these tributaries. PP generally cannot be directly used by plants; however, with the functioning of acid-producing microorganisms, it can be transformed into soluble phosphorus, a state that is storable in water. The soluble phosphorus first combines with some base compounds, and then changes into an insoluble calcium salt, magnesium salt and ferric salt, which constitutes the circulation of phosphorus in tributaries [25,26]. In addition, the PP gradually settles down as the flow rate decreases. When the phytoplankton consumes a large amount of phosphorus, deposited phosphorus is gradually released and forms a new phosphorus balance. DTP is the main form of TP in most of the tributaries, which shows that phosphorus content directly absorbed and used by plants is relatively high.

As a whole, the nitrogen and phosphorus contents in the tributaries are relatively high especially in Sihe River and Shendinghe River, basically more than the critical value (0.2 mg l^−1^, 0.02 mg l^−1^) which makes algae growth easy [27]. Therefore, there are significant security risks of pollution to water quality, such as algae may rapidly grow and propagate, or even exhibit an outbreak of algae water bloom when the water dynamics and other conditions are seriously affected.

#### 3.1.5. Change in nutrient structure

The nutrient structure is often expressed by the N/P ratio [28–30]. DIN is the main nitrogen form that can be used directly; and dissolved inorganic phosphorus is the main phosphorus form that can be used directly. In this study, DTP is used instead of dissolved inorganic phosphorus, and the N/P ratio is the DIN/DTP ratio. When the N/P ratio is greater than 16, phosphorus is the limiting factor of phytoplankton; when the N/P ratio is less than 16, nitrogen is the limiting factor of phytoplankton [31].

As illustrated in table 2, the N/P ratios in Laoguanhe and Hanjiang Rivers are more than 30 in three seasons. It indicates that phosphorus will be preferentially consumed to a low value and become the limiting factor of phytoplankton when algae grow abundantly. Nitrogen is the limiting factor of phytoplankton in Langhe River (in May), Jianhe River (in May), Tianhe River (in December) and Sihe River (in May and August). As a whole, phosphorus is more likely to be the limiting factor of phytoplankton in the tributaries.

**Table 2.** N/P ratio in the tributaries.

	N/P		
tributaries	May	August	December
R1	16	81	53
R2	40	128	60
R3	7	60	95
R4	12	21	23
R5	19	32	103
R6	11	8	18
R7	28	17	25
R8	16	117	38
R9	25	187	13
R10	47	165	36

### 3.2. Cluster analysis for the tributaries

**Figure 7.**
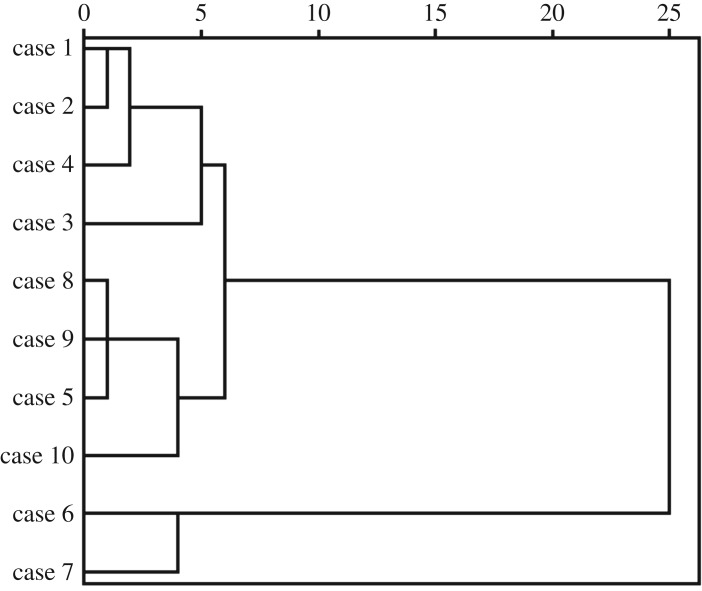
Cluster analysis results for water quality parameters in 10 tributaries.

The water quality parameters in 10 tributaries were analysed by cluster analysis for more targeted governance. As shown in figure 7, 10 tributaries in Danjiangkou Reservoir can be grouped into three types. Type I contains four tributaries (i.e. Danjiang River, Laoguanhe River, Langhe River, Jianhe River) and their water qualities are mainly influenced by non-point sources of pollution because they flow through rural areas. Type II consists of two tributaries (i.e. Sihe River and Shendinghe River) and their water qualities are affected by point sources of pollution because they flow through Shiyan City and receive huge amounts of industrial wastewater and municipal sewage. The remaining rivers are regarded as type III and their water qualities are critically affected by both point and non-point sources of pollutions.

### 3.3. Correlation among water quality parameters

Water quality parameters have different influences on TN and TP. Both TN and TP are significantly correlated with COD_Mn_ and their correlation values are higher than 0.8 with *p* < 0.01. In addition, the positive correlations between TN and EC, Cl^−^, as well as $\text{S}{{\text{O}}_{4}}^{2-}$ indicate that increased levels of these water quality parameters are beneficial to the increasing levels of TN. However, the negative correlations manifest a non-synergistic effect on the existence of each factor. As presented in table 3, correlations between TP and other water quality parameters are similar with that of TN, which implies that TN and TP may have similar pollution sources.

**Table 3.** Correlation among water quality parameters.

	Q	TN	TP	DO	PH	COD_Mn_	EC	F^−^	$\text{S}{{\text{O}}_{4}}^{\text{2}-}$	Cl^−^
Q	1									
TN	−0.312	1								
TP	−0.271	0.876**	1							
DO	0.172	−0.686*	−0.889**	1						
PH	0.363	0.093	0.127	−0.121	1					
COD_Mn_	−0.533	0.831**	0.883**	−0.628	−0.043	1				
EC	−0.389	0.944**	0.833**	−0.689*	−0.019	0.790**	1			
F^−^	−0.653*	0.705*	0.516	−0.319	−0.349	0.685*	0.773**	1		
$\text{S}{{\text{O}}_{4}}^{2-}$	−0.382	0.858**	0.952**	−0.829**	−0.004	0.931**	0.834**	0.654*	1	
Cl^−^	−0.55	0.768**	0.521	−0.473	−0.11	0.516	0.733*	0.761*	0.567	1

*Significant correlation at the 0.05 level (2-tailed).

**Significant correlation at the 0.01 level (2-tailed).

### 3.4. Inputs of total nitrogen and total phosphorus from 10 tributaries

The annual average inputs of TN and TP from the 10 tributaries have been estimated using equation (2.2). As presented in table 4, the annual average input of TN from the 10 tributaries is 63 347.31 t. Among the 10 tributaries, Hanjiang River has the biggest contribution for inputs of TN, which accounts for 68.79%. Duhe and Laoguanhe Rivers also have a big contribution for inputs of TN, which account for 15.38% and 8.71%, respectively. Similarly, the annual average input of TP from the 10 tributaries is 2045.52 t. Hanjiang River accounts for 63.21%, which becomes the biggest contribution for inputs of TP. According to the different types of sources of pollution, the tributaries can be grouped as non-point source controlling rivers (type I), source controlling rivers (type II) and integrated controlling rivers (type III). The contributions of these three types of tributaries for TN input are 10.73%, 0.45% and 85.82%, respectively, and for TP input are 8.61%, 5.92% and 85.47%, respectively.

**Table 4.** The annual average input of TN and TP from the 10 tributaries in units of (t a^−1^).

tributaries	Danjiang River	Laoguanhe River	Langhe River	Jianhe River	Guanshanhe River	Sihe River	Shendinghe River	Duhe River	Tianhe River	Hanjiang River
Q (m^3^ s^−1^)	6.52	60.00	6.71	0.47	3.20	4.12	3.59	187.00	14.00	800.00
W_TN_ (t a^−1^)	392.00	5518.00	840.38	45.70	270.41	948.92	1223.30	9739.71	785.50	43 583.38
W_TP_ (t a^−1^)	7.56	146.95	19.36	2.34	4.75	73.00	48.61	416.82	33.23	1292.90

The significant contributors of TN and TP in the reservoir are the tributaries. According to the results of the inputs of TN and TP, cluster analysis and pollution characteristics, different pollution control measures should be undertaken. The TN and TP contents in Sihe River and Shendinghe River are the highest, while the percentages of input of TN are 1.5% and 1.93%, and the percentages of input of TP are 3.57% and 2.38%, respectively. As the two rivers are polluted by domestic sewage and other point sources, the government should strengthen the construction of urban sewage plants as well as dephosphorization and denitrification projects. For Hanjiang River and Duhe River, whose water quantities are huge, control measures against point source and non-point sources of pollution should be strengthened.

## 4. Conclusion

It can be seen from the presentation above that (i) the concentrations of nitrogen and phosphorus in Sihe and Shendinghe Rivers are higher than those in other tributaries; (ii) TP, TN and DIN are in the forms of DTP, DTN and $\text{N}{{\text{O}}_{3}}^{-}\text{-N}$, respectively, in May, August and December; (iii) compared with nitrogen, phosphorus is more likely to be the limiting factor of phytoplankton; (iv) it was found through cluster analysis that 10 tributaries could be grouped as point source controlling rivers, non-point source controlling rivers and integrated controlling rivers; and the percentages of input of TN in the three types are 10.73%, 3.45% and 85.82%, while the percentages of input of TP are 8.61%, 5.92% and 85.47%, respectively. These findings can provide an important basis for effective measures on safe water provision to Beijing.

## Data accessibility

Our data are deposited at Dryad (http://dx.doi.org/10.5061/dryad.17b01) [32].
